# Predicting evolutionary outcomes through the probability of accessing sequence variants

**DOI:** 10.1126/sciadv.ade2903

**Published:** 2023-07-28

**Authors:** P. Alexander Gunnarsson, M. Madan Babu

**Affiliations:** ^1^ MRC Laboratory of Molecular Biology, Francis Crick Avenue, Cambridge CB2 0QH, UK.; ^2^ Department of Structural Biology and Center of Excellence for Data-Driven Discovery, St. Jude Children’s Research Hospital, Memphis, TN 38105, USA.

## Abstract

Natural selection can only operate on available genetic variation. Thus, determining the probability of accessing different sequence variants from a starting sequence can help predict evolutionary trajectories and outcomes. We define the concept of “variant accessibility” as the probability that a set of genotypes encoding a particular protein function will arise through mutations before subject to natural selection. This probability is shaped by the mutational biases of nucleotides and the structure of the genetic code. Using the influenza A virus as a model, we discuss how a more accessible but less fit variant can emerge as an adaptation rather than a more fit variant. We describe a genotype-accessibility landscape, complementary to the genotype-fitness landscape, that informs the likelihood of a starting sequence reaching different parts of genotype space. The proposed framework lays the foundation for predicting the emergence of adaptive genotypes in evolving systems such as viruses and tumors.

## Teaser

The evolution of protein features is predicted through a method based on the biochemistry of sequence replication.

## INTRODUCTION

Predicting evolutionary outcomes in rapidly evolving systems, such as viruses, is a fundamental problem with major implications for human health and society. Current evolutionary prediction models predominantly involve population modeling, phylogenetics, and the distribution of fitness effects (*1*–*3*). These strategies have been successfully used to predict evolution on the scale of organisms and populations. They have made important contributions to our ability to predict which preexisting viral strains are likely to become dominant and to rationally assist vaccine design by informing which strains to include during vaccine development (*4*). However, these models do not attempt to predict the emergence of new variants at the molecular level that may become important, as they do not explicitly consider the mechanisms of the mutational and molecular processes that drive genetic variation (*5*).

**Fig. 1.**
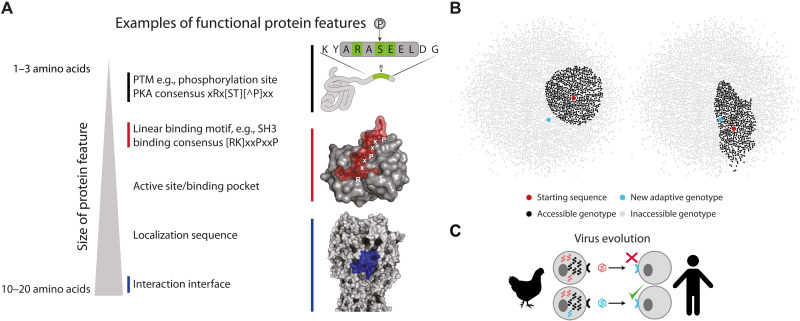
The role of protein features and available genetic variation in adaptation. (**A**) Functional protein features are of different sizes and can be encoded by ensembles of sequences that define the function. Examples include post-translational modifications (PTMs) such as phosphorylation (pictured), glycosylation, and ubiquitination; linear binding motifs including SH3 (pictured), PDZ, and SH2 domains; and more complex features such as interaction interfaces [influenza A virus (IAV) H3 putative binding interface pictured], which are spatially clustered rather than in a linear sequence. (**B**) Sampling genotype space. Only available genotypes can result in adaptation. Whether a genotype is accessible depends on its starting point and how likely the starting genotype is to mutate to a target genotype. The left panel shows a starting sequence that cannot sample a specific adaptive genotype. The right panel shows a different starting sequence in the same landscape that can access that specific adaptive genotype due to the specific biases that determine how easily different sequences are sampled through mutation. (**C**) Viruses can shift hosts through mutations that enable them to bind, e.g., human receptors. This process depends on the emergence of target variants carrying the new functionality through the emergence of a new protein feature (i.e., a single or small number of amino acids that may confer specific function such as binding). This may be predicted if the probabilities of different variants emerging were known.

Variants with new protein features (*6*, *7*) may confer new functions such as altered binding to a host protein or evasion of the immune response, which can be selected during evolution (*8*–*10*). Functional features in proteins (e.g., motifs) are typically mediated by a single or a small collection of amino acids that confer molecular functions such as protein binding or glycosylation sites, phosphorylation sites, and other interaction motifs (Fig. 1A) (*11*). These features are not defined solely by a specific sequence but by an ensemble of short amino acid sequences as defined by their functional attributes. Although protein features are critical for adaptation, unless variants with such features are generated in the first place, evolution cannot select them as adaptations. Predicting their evolution has been considered intractable because of the unpredictable nature of mutational outcomes (*5*). Here, we postulate that determining the probability of the emergence of functional protein variants by considering the mechanistic biases in the mutational processes may enable us to obtain a more accurate estimate of the standing variation that can become available for selection (Fig. 1B). Given that natural selection can only operate on what is available, this approach describes a probabilistic perspective of evolutionary outcomes given a starting sequence, thereby facilitating prediction of the emergence of functional features on the protein level.

We leverage this perspective to define the concept of “variant accessibility,” which is the probability of accessing a set of genotypes encoding protein sequences with a particular functional feature from a starting genotype before selection (e.g., the probability of a viral coat protein gaining the right glycosylation site to evade the host immune response from a current strain or a mutation that may switch host specificity; Fig. 1C). We show that this probability is shaped by the mutational biases of nucleotides during the biochemical process of replication and the structure of the genetic code. Using the influenza A virus (IAV) as a model, we report that relative differences in variant accessibility can play a major role in the emergence of adaptive variants. Contrary to what is commonly assumed, we show that more accessible but relatively less fit variants can often emerge as adaptations rather than the fittest variants, making evolutionary outcomes more predictable. Last, we introduce the concept of the “accessibility landscape,” complementary to the fitness landscape, that informs the likelihood of a starting sequence reaching different parts of the genotype space. In this manner, our work lays the foundation for predicting the emergence of adaptive genotypes in evolving systems linked to infection and disease.

## RESULTS

### Variant accessibility is defined by codon choice and biased mutation rates

**Fig. 2.**
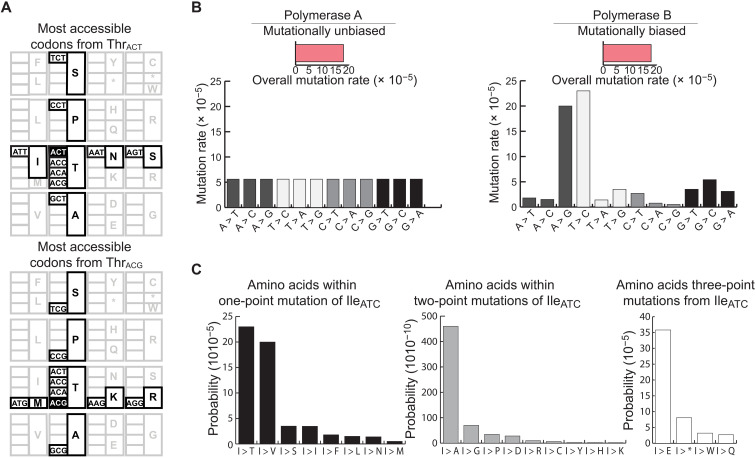
The defining properties of variant accessibility. (**A**) The most accessible amino acids from threonine (Thr) codons ACT and ACG are highlighted. Depending on codon choice, the mutational outcomes of amino acids change markedly due to the structure of the genetic code. ACT can mutate to isoleucine (Ile) through a C > T mutation; however, ACG requires two mutations to reach Ile. (**B**) The same average mutation rate can still manifest extreme biases for individual nucleotide mutation rates. These biases have the potential to markedly affect variant accessibility on the nucleotide and amino acid levels. On the left is a polymerase with no bias, and on the right is the measured mutational bias in H1N1. Despite the differences in bias, both polymerases have the same overall rate, assuming even nucleotide composition. (**C**) The probability of mutating all amino acids from the Ile codon ATC in influenza. This defines a codon’s accessibility profile and shows the large differences in accessibility that result from the structure of the genetic code and the mutation rate biases.

We postulated that one of the principal variables that determine the accessibility of a protein variant is the codon choice in the starting genotype (i.e., the specific nucleotide sequence of a protein-coding region). This results from the way different amino acids are encoded due to different codon choices requiring different numbers of mutations to substitute to a target protein variant. To illustrate the role codon choices can have on accessibility, we considered a hypothetical threonine (Thr) to isoleucine (Ile) (Thr > Ile) variant of hemagglutinin, the key antigenic surface protein of IAV. Such a substitution, Thr^315^Ile, was among the substitutions found to be required for H5N1 to spread between ferrets via respiratory droplets (*12*, *13*). How does codon choice affect the accessibility of Ile, given the choice of different Thr codons in the starting genotype? Because Thr is encoded by ACN (where N is any nucleotide), and Ile is encoded by AUH (where H is U, C, or A). Thr_ACU_ > Ile_AUU_ can happen through a single C > U substitution, whereas Thr_ACG_ > Ile_AUH_ requires two substitutions (Fig. 2A). Considering that the average IAV mutation rate is ~2 × 10^−4^ errors per nucleotide per replication (*14*), Thr_ACU_ > Ile_AUH_ would occur once per ~10^4^ replications, on average, whereas Thr_ACG_ > Ile_AUH_ would occur once per ~10^8^ replications. Codon choices can therefore have a large effect on variant accessibility, as the need for additional mutations markedly lowers the accessibility from a starting genotype.

It is also apparent that any biases in the biochemical rates of specific nucleotide substitutions will further affect the probability of specific changes. Biochemical mutation rate bias depends on the properties of the polymerase and error correction mechanisms, and in most systems, the mutation rates vary among the 12 nucleotide substitution classes (A > C, A > G, etc.) (*15*–*18*). The biochemical rates for all 12 substitution classes have been carefully determined for two IAV strains (H1N1 and H3N2) in fluctuation tests (*14*). This work revealed a strong bias toward U > C and A > G mutations (Fig. 2B and table S1). On the basis of the rates alone, it would be expected that ~70% of all the variants are generated through U > C or A > G mutations, assuming uniform nucleotide distribution in the starting sequence. We then considered the impact of those mutation rate biases on the accessibility of a specific genotype by using the same Thr > Ile example as above. Thr_ACU_ > Ile_AUU_ would occur with a probability of ~10^−5^, and Thr_ACG_ > Ile_AUA_ would occur with a probability of 10^−10^. Thus, compared to the scenario with no mutational rate bias, factoring the experimentally measured mutation rate biases led to a 10- to 100-fold difference in the expected probability of accessing this variant in this example (e.g., a difference of 10^−8^ compared with 10^−10^ for Thr_ACG_ > Ile_AUA_).

**Fig. 3.**
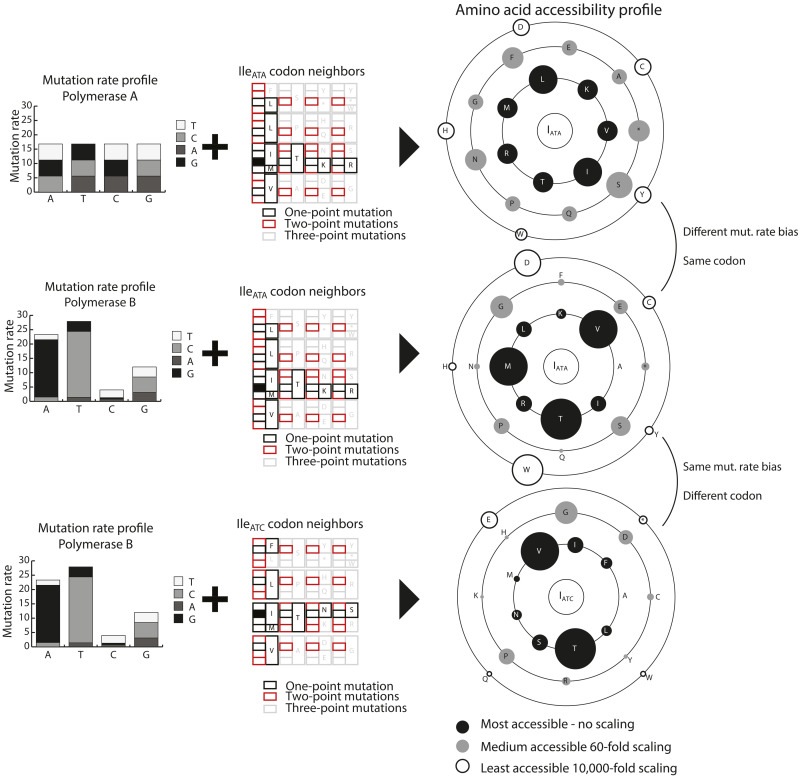
Comparing the effects of mutation rate bias and codon choice on amino acid accessibility by using the accessibility profile as a visual representation. Different rate biases markedly change which amino acids are accessible, as seen when comparing the first two rows. In the top row, leucine (Leu; L) is the most accessible, and all the other residues in the inner circle are equal. When the influenza rate bias is considered (middle row), valine (Val; V), methionine (Met; M), and Thr (T) become the most accessible, and Leu and lysine (K) are much less accessible. Similarly, when encoding the same amino acid with a synonymous codon, it completely changes which other amino acids are accessible and which are not (comparing middle and bottom rows). For example, Met is highly accessible when Ile is encoded with ATA, but it is not accessible when Ile is encoded with ATC, given the IAV polymerase mutation rate biases.

We extended the use of biochemical mutation rate bias and codon choice to define a general framework for quantifying the accessibility of any protein feature, such as phosphorylation or glycosylation sites in IAV sequence evolution. Thus, on the basis of these simple mechanistic observations, we can calculate the probability of accessing a protein feature (e.g., phosphorylatable residue) through mutation. For IAV, we used the experimentally measured biochemical mutation rates (*14*) and determined all the nucleotide mutations that enable a starting codon to substitute to a target set of codons (fig. S1). For example, we determined the amino acid accessibility profile for Ile_AUC_ and represented the accessibility scores as a “satellite plot.” Because of high biochemical mutation rate bias for U > C and A > G in influenza viruses, Thr and valine (Val) were the most accessible amino acids from Ile_AUC_, with probabilities >2 × 10^−4^ per round of replication (Fig. 2C). The other residues within one nucleotide substitution from Ile_AUC_ (serine, Ser; asparagine, Asn; phenylalanine, Phe; leucine, Leu; or methionine, Met) were ~10 times less accessible (probabilities lower than ~3 × 10^−5^). Using the accessibility profile, we compared the effects of both codon choice and biochemical mutation rate bias on the overall amino acid accessibility for specific codons (Fig. 3 and fig. S2). For instance, for Ile_AUA_, in the absence of biochemical mutation rate bias, Leu would be the most likely substitution outcome. However, when the IAV mutational bias was considered, substitutions to Met, Val, and Thr were favored. This approach enabled us to determine the probability of accessing any set of amino acids that define a protein feature from a given protein-coding sequence. In this manner, we can infer the likelihood of accessing a specific protein feature in a population before selection can operate.

### Codon choice and biochemical mutation rates determine the prevalence of the target variant in an evolving population

The sampling of new variants depends on the starting genotype sequence, the size of the initial population, the number of replication cycles, and the number and type of mutations required for the genotype to gain specific protein features (fig. S3, A to C). For a new variant to be successful as an adaptation and become the predominant variant, it must first arise through mutations, i.e., be accessible. The likelihood of fixation of a new variant is additionally dependent on its relative population size, i.e., the fraction of the variant genotype in the total population (*19*). How does the accessibility of a variant from a starting sequence affect its population size in an exponentially growing population? Answering this question may enable us to determine how accessible a new variant would need to be to emerge in sufficient numbers, thereby facilitating survival and spread, such as in the case of viruses or tumor cells.

**Fig. 4.**
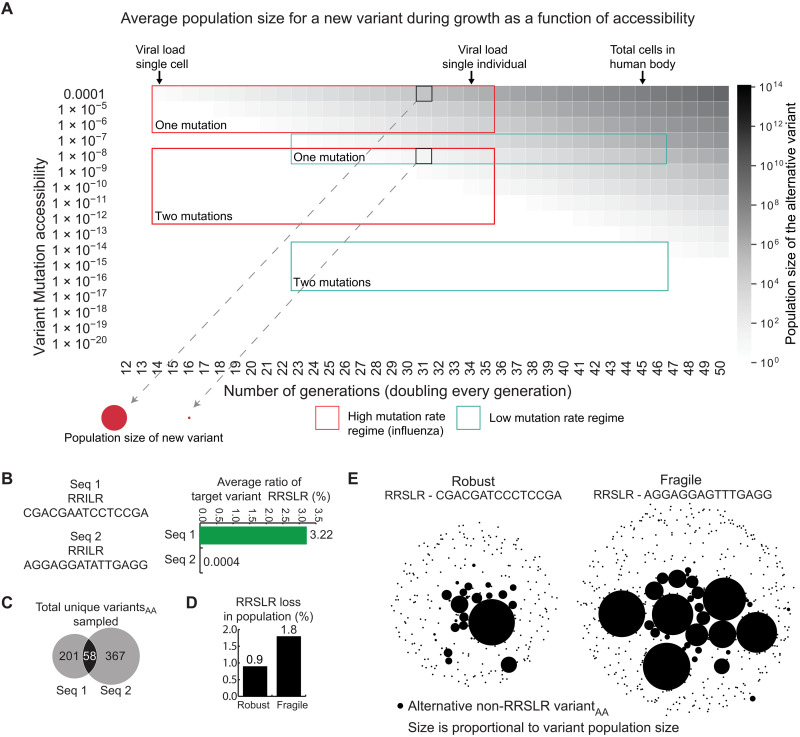
The impact of variant accessibility on the variant population size and sampling of sequence space during growth. (**A**) Each row shows the population size of a growing population of sequences (*x*) and the population size of a variant (heatmap) of different accessibility (*y*) (assuming exponential growth of equal fitness). The red and green boxes indicate the population sizes of viruses and eukaryotes, respectively, and the accessibilities defined by one to two mutations. The black boxes show the populations of virus variants needing one or two mutations, highlighting that a variant within one nucleotide substitution makes up a subpopulation of ~10,000 virus particles. In contrast, a variant protein within two susbtitutions will rarely be seen. Similarly, in the number of replications expected in human cells, two specific point mutations are highly unlikely, as highlighted by the second green box, due to the low average mutation rates. (**B**) Simulation results of two variants with identical amino acid sequences encoded using different codons at the nucleotide level. Seq 1 readily samples the target RRSLR, and it comprises a substantial subpopulation during growth. During the growth of Seq 2, RRSLR is extremely rare and barely emerges. (**C**) The two sequences in (B) also sample disparate regions of sequence space during simulations. By using different codons, different variant sequences are accessible. As a result, Seq 1 samples only 259 alternative amino acid sequences, and Seq 2 samples 425 amino acid sequences, of which 58 are shared. (**D**) The loss of the protein feature in a starting sequence during growth. Identical peptides encoded by different codons lose the starting variant to different extents, depending on the codon choices and mutational biases. (**E**) The target sequence spaces starting from the fragile and robust sequences, respectively. The fragile starting sequence generates many more variant sequences and in larger numbers. This increased rate of loss leads to fewer descendants of the fragile variant, which reduces fitness.

To answer this question, we first investigated the probability of accessing different target variant sequences from a given starting sequence, with accessibility values ranging from 10^−4^ to 10^−30^ (Fig. 4A). These accessibility values represent the range of expected accessibilities from the highest in a single-nucleotide substitution in a virus (~10^−4^) to a scenario with three low-probability substitutions (~10^−27^). During an infection, the typical viral population size in an individual is ~10^10^ virus particles (*20*). A variant requiring a single nucleotide substitution with an accessibility of 10^−4^ would, on average, have a population of ~10^5^ particles in an infected individual, which is only a small fraction of a percent of the total viral population (Fig. 4A). In contrast, a variant requiring two nucleotide substitutions with an accessibility of 10^−11^ would, on average, not be sampled at all. To put it differently, the less accessible variant would only emerge, on average, as a few copies in 10 of 100 individuals, while the more accessible variant would, on average, make up 10^5^ viral particles in all 100 individuals, making it much more likely to spread further.

We next investigated the importance of codon choice in the probability of sampling a protein feature and consequently the expected prevalence of variants in a population. This is important, because, in viruses, many related strains circulate, often encoding genes with several silent mutations resulting in slightly different codon usage. To what extent can these differences determine whether synonymous sequences evolve a new protein feature? To address this, we examined the emergence of a new phosphorylation site: In viruses, phosphorylation sites regulate many aspects of the infection cycle, and adaptation through the evolution of phosphorylation sites is common (*21*). The peptide sequence RRILR (arginine-arginine-isoleucine-leucine-arginine) is one amino acid substitution away from the phosphorylation motif recognized by protein kinase A, RRSLR (described by the regular expression [RK][RK]x[ST][^P]x). Starting sequences that are near a motif consensus may easily gain a substitution, thereby enabling them to acquire new functions de novo.

We considered two synonymous versions of RRILR using alternative codons for all amino acids, RRILR_1_ and RRILR_2_ (see Fig. 4B for the specific genotype). RRILR_1_ can sample RRSLR with a single-nucleotide mutation, whereas RRILR_2_ required two nucleotide mutations; thus, RRSLR is ~10,000-fold more accessible from RRILR_1_ compared to RRILR_2_. We simulated digital populations of these sequences, replicating and mutating according to the dynamics in IAV infections (Materials and Methods) (*22*, *23*). Populations of RRILR_1_ sampled RRSLR frequently, and it emerged as a relatively large proportion of the replicating population. In contrast, populations of RRILR_2_ did not sample RRSLR in substantial numbers under the parameters explored (Fig. 4B). The importance of codon choice was underscored by the observation that the sequence space (at the amino acid level) sampled by the two sequences was largely different, with only a small amount of overlap (Fig. 4C and fig. S4). We also compared two nonsynonymous sequences, RCFLR and RTSLR, and illustrated their mutational paths to a target sequence RRSLR (fig. S3C). Despite RCFLR requiring two amino acid substitutions, it counterintuitively gained the nucleotide substitutions required to access RRSLR much more easily, because the codon choices and IAV nucleotide biases made them much more accessible.

We also investigated the effect of codon choice and biochemical mutation rate bias on the likelihood of losing an existing protein feature, i.e., mutational robustness. Using the motif RRSLR as the starting sequence, we determined how often this phosphorylation motif can be lost through mutations leading to amino acid substitutions. We again considered two synonymous sequences, RRSLR_robust_ and RRSLR_fragile_, and simulated a digital population using the mutation rate bias determined for IAV. As expected, on the basis of the model calculations for the codons chosen, RRSLR_robust_ was substantially more robust to mutations than was RRSLR_fragile_, which lost the motif at twice the rate of RRSLR_robust_ (Fig. 4D). This results from different sampling of the genotype landscape and the robust sequence having more access to synonymous sequences and being encoded by specific nucleotides with lower mutational rates due to the mutational biases. If the motif sequence is critical for survival, then we would expect the growth rate of a population using RRSLR_fragile_ to be lower than one using RRSLR_robust_, since a more substantial number of individuals in the former would lose the protein feature during replication, resulting in fewer successful descendants (Fig. 4E). To fully understand mutational robustness, it is also important to have an accurate understanding of which amino acids are tolerated in the motif, i.e., how redundant the motif is. A more redundant motif will be less affected by these biases since many mutational outcomes can be tolerated. When rerunning the simulations for a more degenerate motif sequence [RK][RK][ST]xx where x is any amino acid, the robust sequence had a smaller, but still substantial advantage over the fragile sequence, as the fragile sequence lost the motif 33% more than the robust sequence. The exception where redundancy will remove the role of biases completely is when all possible mutational outcomes have the same result, such as a position that can tolerate [FLIMV], where any mutation at position 1 or 3 is silent, and all codons in the group share the same nucleotide at position 2.

In summary, despite identical amino acid sequences, codon choices and overall accessibility due to biochemical mutation rate biases determine the sequence space that is sampled in the population in a predictable way. Thus, codon choices can aid in the prediction of specific outcomes that may be adaptive. When the frequency of specific mutation classes, such as T(U) > C, is disproportionately high, the effects on variant accessibility for some amino acids will be highly skewed. Thus, using different codons can result in markedly different accessibilities for certain adaptive variants and can influence the exploration of the sequence space before selection.

### Variant accessibility can be more important for adaptive outcomes compared to relative fitness

**Fig. 5.**
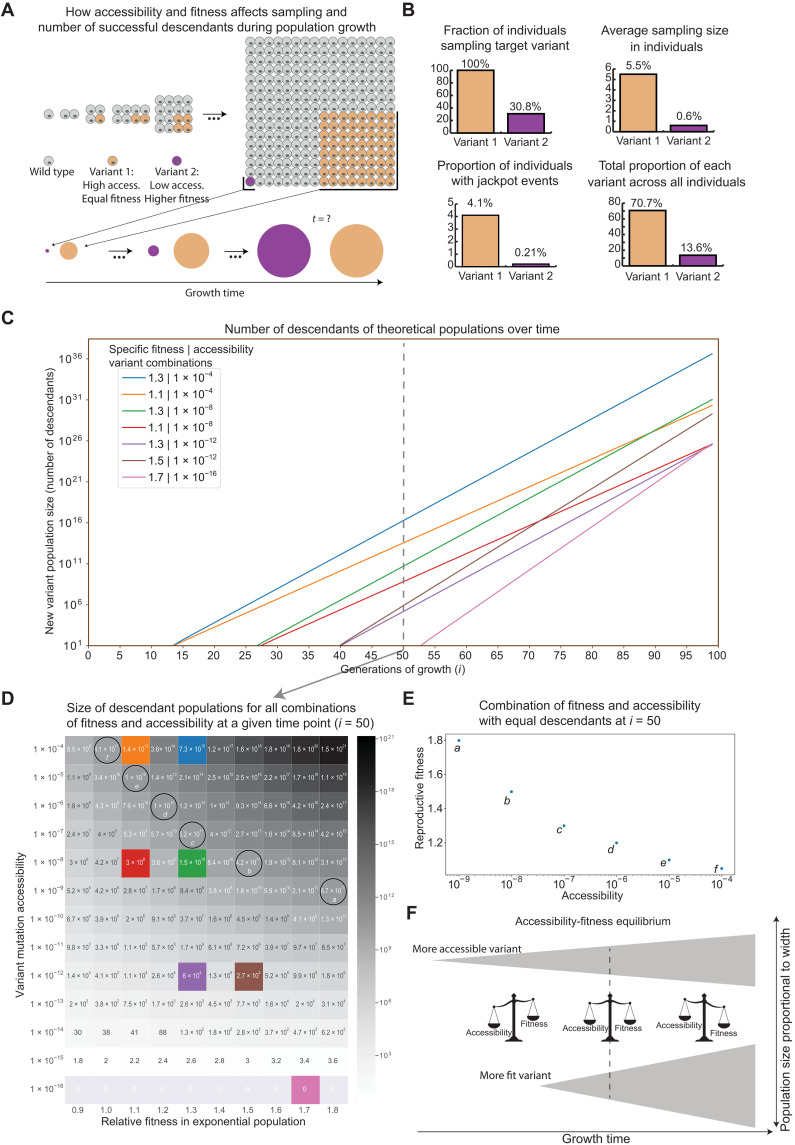
Accessibility and fitness of new variants in a growing population. (**A**) More accessible variants emerge earlier compared to less accessible new variants. Their relative fitness describes how well they grow after they emerge. For a given fitness and accessibility, there is a time point, *t*, when they become equivalent in size and thus have equal apparent fitness. The success of different variants depends on accessibility, fitness, and the biological time scale under which the variant operates. (**B**) Accessibility versus fitness simulations. Target variant 1 with an accessibility of 2.3 × 10^−4^ and a relative fitness of 1.2 emerges as an adaptation in most simulation runs, compared to variant 2 with an accessibility of 5 × 10^−6^ and a relative fitness of 1.4. The variants require the substitutions Ile > [Thr or Ser] (high probability [T > C or T > G]) or Ile > Met (low probability C > G), respectively. (**C**) Comparison of seven combinations of fitness and accessibility over time. The more accessible the variant, the earlier it emerges. The higher the fitness, the faster it grows. Relative fitness needs to be very high for the more fit population to catch up in a reasonable time frame, even for small differences in accessibility equivalent to a single additional mutation. (**D**) Comparison of variant population sizes for combinations of fitness and accessibility at 50 generations of growth (doubling each generation). The combinations of accessibility and fitness presented in (C) are highlighted as colored squares. In addition, the circles highlight the equal variant populations at the accessibility-fitness equilibrium as plotted in (E). (**E**) Combinations of equivalent variant population sizes at generation 50, indicate that a highly accessible variant would do as well as a variant with high relative fitness. (**F**) The accessibility-fitness equilibrium. To the left of the equilibrium, the more accessible variant will be more successful due to larger population numbers. To the right of the equilibrium, fitness becomes the dominant factor determining the population size of the variant.

While accessibility informs the likelihood of sampling a particular variant, the fitness of a variant in a specific environment is determined by whether it promotes survival and successful reproduction (fig. S5). The fitness of a variant is defined as its reproductive success over time, i.e., the number of descendants with that variant at a given point in time. The relative fitness of two existing variants in a population is thus simply the ratio of descendants of those two variants. However, when considering the fitness of two new variants that have yet to emerge in the population, the number of their descendants at a given future time will depend on how early and how often the variants initially arise as well as their reproductive success once they are in the population (Fig. 5A). Hence, the apparent relative fitness of new potential variants will be a function of both accessibility and reproductive success.

To better understand the relative roles of accessibility and fitness, we computationally simulated viruses infecting cells in individuals in a population (Materials and Methods) (*22*, *23*). Infected cells were defined as populations of 10^4^ “viral” sequences grown from a single founding sequence [wild-type (WT)], mutating according to the experimentally determined biochemical mutation rates for IAV (fig. S6). An infected cell can then infect additional cells within an individual according to the fitness of its variant viral sequences. After ~5000 cells were infected in this manner in one individual, viral sequences were selected to infect new individuals according to their fitness. We simulated a total of ~10^11^ sequences across ~5,000,000 cells in 1000 individuals and determined how often specific mutations resulting in target genotypes with defined protein features emerged in individuals and the number of cells with target genotypes in each individual (fig. S6). We also assessed the number of jackpot events, i.e., chance mutations that arise early in replication and individual infections, resulting in >25% of all “viral particles” in an individual being the target genotype instead of the WT sequence. Jackpot events are important for adaptation to new selection pressures (*24*); therefore, we determined how often such events occurred and quantified their impact on the prevalence of the variant sequence.

We first simulated a sequence encoding RRILR (WT) and assessed the emergence of two alternative variants, RR[TS]LR, where the brackets indicate that either Thr or Ser are permissible (variant 1) and RRMLR (variant 2; see Materials and Methods for details and for nucleotide sequences). Both variants are a single nucleotide mutation away from the WT sequence, but because of mutation rate biases and the choice of codons, the probability of accessing variant 1 is 2.3 × 10^−4^ per replication, approximately 50 times higher than that for variant 2, which had an accessibility of 5 × 10^−6^. To explore a specific regime where relative fitness differences are large, we chose to set the relative fitness of variant 1 to 1.2 compared to the WT and that of variant 2 to 1.4. In the simulations, the initial WT population evolved to variant 1 almost exclusively, whereas variant 2 rarely emerged. Thus, the highly accessible variant of lower fitness predictably and consistently emerged and got fixed in the population instead of the less accessible variant of higher fitness, which rarely emerged at all (Fig. 5B). We noted that the accessibility difference between variants 1 and 2 was relatively small, while the relative fitness difference was larger than those found in nature (*25*). For context, in a study measuring the fitness effects of beneficial mutations in different bacteriophage genotypes, the average variant had a relative fitness <1.05, with the largest effect being 1.3 (*26*). With lower fitness differences than those used here and larger accessibility differences, the importance of accessibility would only be higher.

In the simulation, variant 1 reached fixation largely due to its high average population sizes and frequent jackpot events resulting from its higher accessibility. On average, variant 1 made up 5.5% of the total viral particles in individuals compared to 0.6% for variant 2 (Fig. 5B). Variant 1 emerged during jackpot events in 4.1% of individuals, and variant 2 emerged in 0.21% as jackpot events, meaning that variant 1 more often comprised >25% of all viral particles in an individual. These properties enable individuals to spread variant 1 more easily (Fig. 5B). Although jackpot events resulting in variant 1 were only seen in 4.1% of individuals, they went on to infect 35% of the subsequently infected individuals, highlighting the disproportionate importance of jackpot events in variant 1 reaching fixation. This observation is in line with early observations on plaque formation by Luria and Delbrück (*24*). Thus, small differences in accessibility can contribute disproportionately to the large differences in population sizes of highly accessible but lower fitness variants in part due to the increased likelihood of jackpot events. We, therefore, concluded that variants of higher accessibility can result in a higher number of successful descendants compared to variants of higher relative fitness but that are not easily accessible.

To further explore the relationship between accessibility and relative fitness, we numerically analyzed a range of values for each parameter. We compared hypothetical new variants of several combinations of accessibility and fitness values in exponentially growing conditions to assess when, on average, they would emerge and start growing and when higher relative fitness (higher growth rate) would result in variant populations overtaking the lower fitness variant populations (Fig. 5C). The advantage of emerging early due to high accessibility resulted in high population numbers for extended periods of growth. The relative fitness needed to be substantially higher in the less accessible populations to catch up, for instance, in the time frame of seasonal virus infections (see Materials and Methods). To compare the impact of different variant accessibility values alongside different relative fitness values, we examined a specific time point that was representative of viral populations during human infection and plotted the average number of descendants (Fig. 5D). This enabled us to define an accessibility-fitness equilibrium, i.e., a point of equivalence between the accessibility and fitness values that would result in approximately equal numbers of descendants for a population at that point of growth (Fig. 5, E and F). Under these simulation conditions, a variant with an accessibility of 10^−5^ and a relative fitness of 1.1 would be as successful as a variant with an accessibility of 10^−8^ and a relative fitness of 1.5. This suggests that higher accessibility results in increased apparent fitness of potential variants because early emergence in a growing population resulted in more progeny as the population grew in these conditions. It is still likely that, in rapidly evolving viruses under strong selection pressures during infection, more inaccessible variants of exceptionally high fitness will occasionally emerge due to rare jackpot mutations. However, the accessibility-fitness balance describes what we expect the evolutionary outcome to be on average across many replicating viruses in many hosts over time. These observations thus indicate that variant accessibility can be a key factor in predicting the emergence of highly accessible protein features as evolutionary adaptations in growing populations.

### Adaptive mutations observed in IAV are highly accessible, and functional protein features use robust codons

**Fig. 6.**
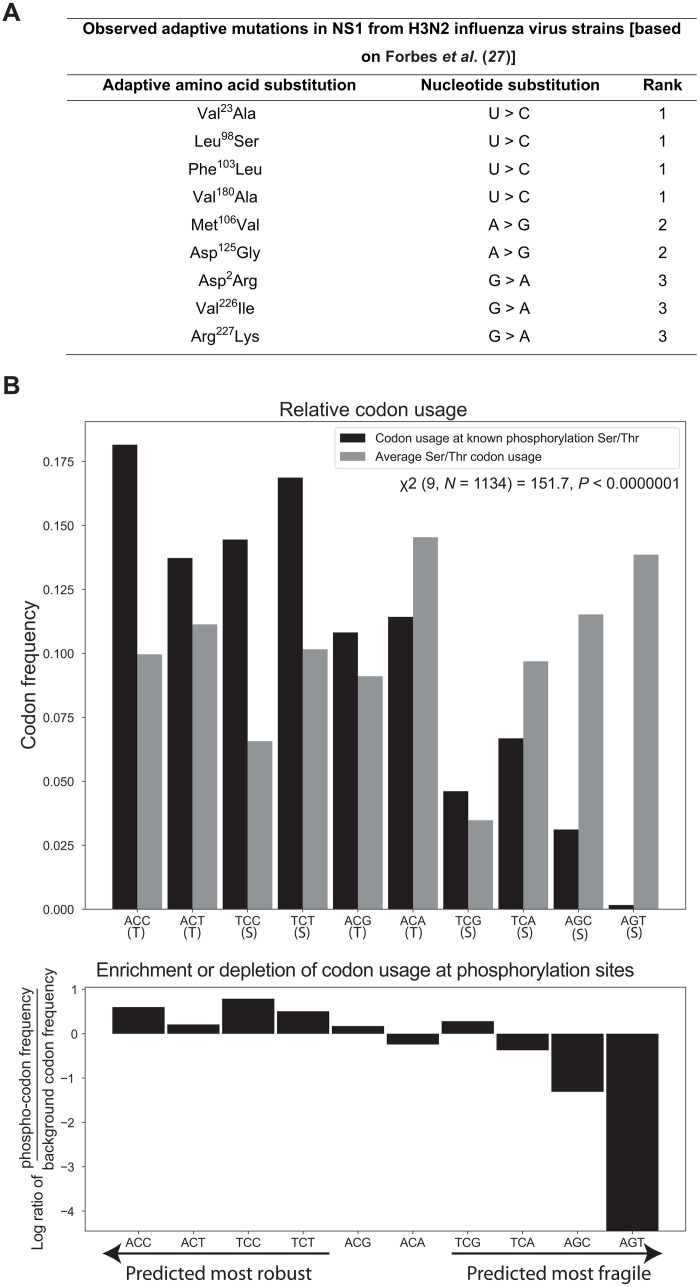
Consequences of accessibility in IAV evolution. (**A**) Nine specific nucleotide mutations that resulted in adaptive evolution in IAV protein NS1 in H3N2 strains are shown alongside the amino acid substitutions. The mutation rates for these mutations are the top three highest (most accessible) in H3N2, as shown in table S1. This highlights the role of accessibility and mutation rate bias in adaptive evolution in H3N2. (**B**) The relative codon usage at Ser/Thr positions of conserved, functionally phosphorylated (phospho) residues is compared to background Ser/Thr codon usage at nonphosphorylated positions. There is a significant difference in the codon usage at phosphorylated Ser/Thr sites [χ^2^ (9, *N* = 1134) = 151.7, *P* < 1 × 10^−7^]. This is driven by enrichment in the usage of more robust codons (Ser_TCC_, Ser_TCT_, Thr_ACC_, and Thr_ACT_), as determined by their calculated loss probability (table S4), and a significant depletion of the much less robust codons Ser_AGC_ and Ser_AGT_ in conserved, phosphorylated Ser/Thr positions [χ^2^ (2, *N* = 1134) = 62.8, *P* < 2 × 10^−14^]. This highlights that more robust, less mutationally sensitive codons (which can be predicted as the accessibility of nonfunctional variants from the starting functional genotype through mutation biases) are selected at key functional sites.

If accessibility plays an important role in evolutionary adaptations, then we expect most adaptive mutations in IAV to be highly accessible. Given the measured mutation rate bias for IAV, the predominant mutations should be U > C or A > G. To explore whether this is the case, we identified key adaptive mutations in nonstructural protein 1 (NS1) in H3N2 strains of IAVs from the literature and inferred the underlying nucleotide mutation based on the codon table (*27*). Because the adaptations are reported as amino acid substitutions, we focused on nine mutations for which codon/nucleotide substitutions were unambiguous. All nine adaptive mutations identified here were the result of a highly likely, accessible mutation (Fig. 6A). As expected, the majority (66.7%) resulted from U > C or A > G mutations; the remaining three occurred through G > A mutations, which is the most accessible after U > C and A > G (Fig. 6A). These findings are consistent with the idea that accessibility is a central parameter influencing evolutionary adaptation.

We then identified a set of functional phosphorylation sites in IAV proteins as reported in the literature (*21*, *28*–*31*). We only included high-confidence, functionally relevant phosphorylated sites as defined by high amino acid conservation across IAV strains, observed phosphorylation reported in the literature, and observed functional and fitness consequences of mutating the site (Materials and Methods) (*21*, *28*–*31*). We analyzed 10,000 strains and identified 31 high-confidence phosphosites across six IAV proteins. On the basis of the simulation results for the robustness of protein features, we expected these sites to be encoded by more robust codons, as they are less likely to be lost due to mutations during replication. Because of the diversity of phosphorylation motifs, we specifically focused on codon usage at the Ser/Thr position, which is well-defined in all these sites and is the residue that is chemically modified. According to the model, we expected Ser codons TCC and TCT and Thr codons ACC and ACT to be favored as well as Ser codons AGT and AGC to be selected against, as they are predicted to be the least robust given the IAV biochemical mutation rates. Correlating the sequence records (nucleotide sequences) with the phosphorylation status on the protein level, we found that usage of less robust AGT and AGC codons was depleted at these Ser/Thr positions, while usage of highly robust TCC, TCT, ACC, and ACT codons was significantly enriched (*P* < 2 × 10^−14^, χ^2^ test; Fig. 6B). This indicated that more robust codons are selected in highly conserved, functionally relevant phosphorylation sites. Together, these observations highlight that both robustness and evolvability are shaped by the accessibility of protein variants given a specific starting genotype through the effects of codon choice and biochemical mutation rate bias.

## DISCUSSION

We have defined and explored the importance of variant accessibility as a key factor for evolutionary adaptation through the gain or loss of protein features. Small differences in the starting nucleotide sequence can markedly change the variant accessibility of target protein features and, by extension, adaptive phenotypes. On the basis of these findings, we propose that the accessibility of a variant from the starting genotype is critical for its likelihood of becoming a successful adaptation. Furthermore, our work highlights that the mechanistic factors that define accessibility can also describe the robustness of protein features. The accessibility model quantifies this robustness and can highlight protein features that are encoded more robustly, with implications for their functionality and fitness (e.g., those encoding a phosphorylation site; Fig. 6B). Because variant accessibility can be determined from the starting sequence and system-specific biochemical mutation rate biases, we anticipate that this model of evolutionary accessibility can be used to predict adaptations in evolving systems. This notion is supported by the finding that several known molecular adaptations in NS1 occurred through highly accessible substitutions (Fig. 6A). By combining accessibility with other models that define the fitness impact of mutations on the structure and function of proteins, we anticipate that prediction of evolutionary outcomes can be greatly improved.

Our work joins the growing body of literature that has shown that biases in the way in which variation is generated in cancers and organisms affect evolutionary trajectories, resistance mechanisms, and adaptations (*32*–*37*). They lend further evidence to the importance of the concept of accessibility during evolution. These studies further highlight the need for a more general conceptual framework to define accessibility and better understand the mechanistic aspects of how such a framework influences the evolution of protein features in these systems, as presented here.

**Fig. 7.**
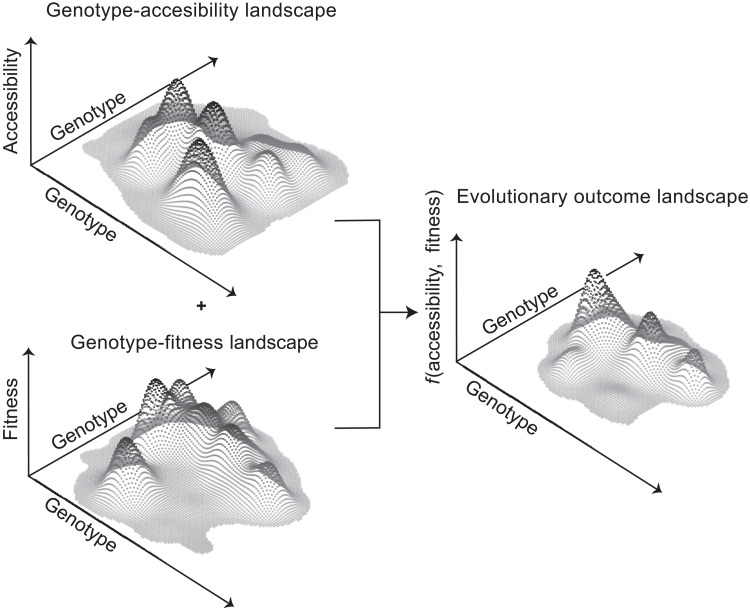
Conceptualizing the genotype-accessibility landscape and the evolutionary outcome landscape. The accessibility landscape describes the probability of a given starting sequence mutating to other genotypes. The genotype-fitness landscape depends on the environment and describes the successful reproduction of a given genotype. Together, these two conceptual landscapes describe the most likely evolutionary adaptive trajectories in organisms. We hypothesize that the accessibility landscape plays a critical role in adaptation and can be used to predict the evolutionary outcome in terms of the emergence of specific protein features, such as a pathogen or tumor cell gaining resistance mutations during treatment.

With these observations in mind, we propose an extension of the concept of the genotype-fitness landscape by presenting a complementary genotype-accessibility landscape. While the fitness landscape is a function of genotype and environment (*38*), the accessibility landscape is a function of a given sequence determined by the mechanistic properties of replication through mutation biases, error correction mechanisms, and the structure of the genetic code. The predictability of evolutionary outcomes is thus a function of both accessibility and fitness (fig. S7). By combining the accessibility and fitness landscapes, we can describe the evolutionary outcome landscape for a starting sequence, which highlights the most likely adaptive evolutionary destinations in genotype space as a function of both variant accessibility and fitness (Fig. 7 and fig. S7). This framework postulates that by determining accessibility and fitness, we can create more accurate predictive models for evolutionary adaptations. Such accessibility landscapes can be determined by in-depth experimental characterization of polymerase mutation biases and error correction biases during replication (*14*, *39*). In combination with detailed quantification of fitness effects of protein variants through approaches such as deep mutational scanning (*40*, *41*), we anticipate that it will be possible to quantify the evolutionary outcome landscape. In this manner, the framework described here can be used to address a range of problems pertaining to evolving systems, including in organismal evolution, the emergence of variants resistant to vaccines and drugs, as well as in cancer and viral evolution.

## MATERIALS AND METHODS

### Variant accessibility framework calculations

We defined variant accessibility as the probability of a starting sequence gaining a protein feature after one round of replication. A protein sequence variant is defined by sets of allowed amino acids at each given position, such as [RK][RK]*x*[ST][^P]*xx*, where *x* is any amino acid, each set of brackets defines a single position, and the residues within brackets are the tolerated amino acids at that position for a defined functional variant. To calculate this probability for a given initial sequence (*Seq*_init_) encoded by *i* codons, we calculated the combined probability of each initial codon being equal to a codon that encodes an amino acid in the defined set after a round of replication. Formalizing this, for a motif with *i* consensus positions, we define a set of key positions as *M_i_* that describe the protein feature as follows

$$M_{i}=\{i\in \{{\mathrm{VariantPos}}_{\mathrm{defined}}\}\}$$

We define a set of codons, *C^i^*, for each position that encodes the allowed amino acids in each position for the variant function of interest

$$C^{i}=\{N_{1}^{i},N_{2}^{i},N_{3}^{i}|AA(N_{1}^{i}N_{2}^{i}N_{3}^{i})\in \{AA_{\mathrm{variant}}^{i}\}\}$$

where *N*_1_, *N*_2_, and *N*_3_ are nucleotides at positions 1, 2, and 3, respectively, in the codon, and the $AA_{\mathrm{variant}}^{i}$ is the set of amino acids allowed at position *i* in the motif sequence. We can thus express the probability of the *i*th codon to substitute to any of the allowed codons as the sum of all paths of substitutions that result in one of those codons

$$P(C^{i}|S^{i})=\sum_{N_{1}^{i},N_{2,}^{i}N_{3}^{i}\in C^{i}}\;P(n_{1}=N_{1}^{i}|n_{1}=S_{1}^{i})*P(n_{2}=N_{2}^{i}|n_{2}=S_{2}^{i})*P(n_{3}=N_{3}^{i}|n_{3}=S_{3}^{i})$$

where $S^{i}=S_{1}^{i}S_{2}^{i}S_{3}^{i}\in {Seq}_{\mathrm{init}}^{i}$. For the whole sequence, the probability of mutating from *Seq*_init_ to the defined variant sequence thus becomes a single-site factorization over all the positions

$$P(\mathrm{variant}|{Seq}_{\mathrm{init}})=\prod_{i\in M_{i}}P(C^{i}|S^{i})$$

### Numeric analysis of variant emergence

We determined the prevalence of theoretical variants with given accessibilities by defining simple growing populations, starting from a single sequence and doubling through replications during every generation. When the sequence is copied, the accessibility describes the probability of the sequence mutating into a theoretical target variant. On average, a sequence with accessibility of 10^−4^ will be sampled once over 10,000 replications. We thus add *n × a* new variants to the replicating population during every round of replication, where *n* is the size of the population (i.e., the number of sequences being replicated) and *a* is the current variant accessibility being probed. In addition, all existing sequences are replicated every round, meaning that the new variant sequences are also growing exponentially after emerging (doubling every round of replication).

### Evolvability simulations

To simulate the emergence of new variants from a starting sequence, we defined two initial starting sequences

1) Seq 1: CGACGAATCCTCCGA encoding RRILR

2) Seq 2: AGGAGGATATTGAGG encoding RRILR

The codons were selected according to their calculated accessibilities to the target sequence (see above). We wrote a custom simulation algorithm that replicates these sequences over time with the mutational probabilities, as outlined in table S1. We replicated sequences using these parameters exponentially until the population of sequences reached ~400, reflecting the number of RNA particles of individual transcripts generated during influenza infection in an individual cell (*22*, *23*). After that, replication continued from the pool of 400 sequences linearly, until the population reached 10^4^ (number of virions in single cells), as this approximates the replicative dynamics during influenza infection. However, the outcome is not sensitive to the specific method of replication and growth, e.g., when compared to an assumption of fully exponential growth to 10^4^ sequences (cf. Fig. 4A). We simulated a total of 10^5^ “cells” or a total of 10^5^ × 10^4^ sequences and quantified the number of variants and the exploration of variant space in this pool of sequences.

### Robustness simulations

Robustness simulations were conducted using the same simulation algorithm as evolvability simulations. Two differently encoded, synonymous sequences were simulated

1) Seq 1: CGACGATCCCTCCGA encoding RRSLR

2) Seq 2: AGGAGGAGTTTGAGG encoding RRSLR

We simulated populations of these sequences and determined the number of sequences encoding the RRSLR protein sequence and the number of sequences encoding other non-RRSLR sequences, meaning that the functional sequence had been lost. In these simulations, if any position mutated to a nonsynonymous sequence, then the motif was considered lost.

### Accessibility and fitness simulations

To simulate the evolution of sequences under viral growth conditions with fitness acting on specific variants, we defined the WT sequence and two variant sequences. The WT starting sequence was as follows

1) WT: CGACGAATCCTCCGA encoding RRILR; relative fitness: 1.0

2) Variant 1: Any nucleotide sequence encoding RR[TS]LR; relative fitness: 1.2

3) Variant 2: Any nucleotide sequence encoding RRMLR, relative fitness: 1.4

All other sequences were assigned a relative fitness of 0.5. The results were similar when the other sequences were assigned a relative fitness of 1.

Cells were then founded by one copy of the WT sequence and replicated using the same algorithm, as described in the previous section, to a size of 10,000 sequences. The fitness variable was used to select sequences to infect new cells for the next generation. Individual sequences were selected at random, with a relative probability of being selected according to their relative fitness. The absolute fitness of the WT sequence was two, so that a cell of only WT sequences would infect, on average, two descendant cells. Thus, a variant of relative fitness of 1.5 would, on average, infect three descendant cells. Starting with one sequence in one cell, we simulated growth to ~5000 cells that was defined as one individual. All the virus particles in an individual then went through another selection step, in which individual sequences were selected to infect new individuals based on relative fitness. Again, WT sequences, on average, infected two individuals. We repeated this pattern of growth and selection over 10 to 13 generations, resulting in ~100 infected individuals, at which point a variant had been fixed in the majority of simulation runs. We also performed independent repetitions of these simulation conditions for 10 replicate simulation runs. In total, we characterized the replication of 10^11^ sequences, over ~5,000,000 cells in ~1000 individuals. This is a smaller-scale simulation that mimics the viral dynamics of growth in single cells, then spread among cells in an individual, and lastly spread to new individuals, where fitness determines the spread among cells and the number of subsequent infections caused by a specific variant (see fig. S6).

### Numeric analysis of accessibility and fitness

To numerically characterize the relative impact of variant accessibility and fitness in exponentially growing populations, we performed calculations on theoretical sequence populations by using combinations of accessibility and fitness values (i.e., relative fitness values: 0.9 to 1.8 and accessibility values: 10^−4^ to 10^−16^). These ranges represent the biological range of accessibility values for one to three mutations. Populations started with one sequence and doubled every round of replication. A new variant emerged through a function that used random number generation that was compared to the predefined accessibility of the theoretical variant to retain the stochasticity of mutational events. A new variant emerged if the random number (between 0 and 1 with over 13 significant figures) was smaller than the probability of variant emergence (e.g., 0.0001), which provides a simple probabilistic function that approximates the mutational events. When a new variant emerged, as determined by the function, it exponentially replicated according to its fitness. The exponential growth rate is therefore higher for variants of higher fitness. Different combinations of accessibility and fitness were compared to analyze the relative importance of early emergence and growth rate on the number of descendants.

### Determination of codon usage bias at phosphorylation sites in IAV

To characterize codon usage at conserved phosphorylated Ser/Thr positions, we first identified functional phosphorylation sites described in the literature. We only kept sites with high conservation across IAV strains and those shown in the literature to be functionally phosphorylated (*21*, *27*–*30*, *41*). We identified 31 phosphorylation sites across IAV proteins NS1, NS2, M1, M2, nucleoprotein (NP), and polymerase acidic (PA) (data S1). Sequence alignments were downloaded from the dataset of a previous publication (*42*). The sequences were filtered to remove ambiguous nucleotide positions, identical sequences, and gapped sequences. The final dataset was as follows: 8663 sequences of NS1 and NS2, 9921 sequences of M1 and M2, 9592 sequences of NP, and 10,651 sequences of PA. These alignments were translated, and the codon usage at each Ser/Thr was identified. We then separated the Ser/Thr sites into two datasets, the phosphorylation sites identified in the literature and all other Ser/Thr sites. We determined the codons used in all strains for these Ser/Thr positions and summarized the codon usage at the phosphorylated positions and the background codon usage at other Ser/Thr sites. We normalised the comparison for each Ser/Thr site based on the conservation level such that if, e.g., position 48 in NS1 only encoded Ser/Thr in 5000 of 8663 strains and 1000 of those used codon TCC, then the frequency of that codon at that position contributed one instance of a ratio of 0.2 (i.e., 1000/5000). To assess the statistical significance of the observed codon usage difference we used a contingency table (table S3), thereby determining what is the likelihood of seeing the observed codon usage across the 31 phosphosites given the background distribution of Ser/Thr positions. The statistical significance was then determined using the chi-square test. We additionally tested whether the usage of robust codons was significantly enriched compared with fragile or neutral codons by grouping the codons according to their loss probability (table S4). Loss probability was calculated using the previously described probability calculation for Ser/Thr codons to mutate into any other non-Ser/Thr codon, with an additional loss penalty for the probability of gaining a stop codon. The top four codons in the table were considered robust, the subsequent four as neutral, and the last two as fragile. The counts of these are represented in a contingency table (table S2). We tested the significance with chi-square with a false discovery rate correction for multiple testing, with the following outcome: (“fragile,” “neutral”), *P* = 1.26098528041 × 10^−11^, corrected: 1.89147792061 × 10^−11^; (fragile, “robust”), *P* = 3.6581805706 × 10^−15^, corrected: 1.09745417118 × 10^−14^; and (neutral, robust), *P* = 0.208947253634, corrected: 0.208947253634.
