# Supplementary material for: Predicting evolutionary outcomes through the probability of accessing sequence variants
Source: Sci Adv. 2023 Jul 28;9(30):eade2903. doi: 10.1126/sciadv.ade2903 (PMC10381947; doi:10.1126/sciadv.ade2903)
Supplement: Supplementary file 1 — Figs. S1 to S7 Tables S1 to S4 Data S1 [file sciadv.ade2903_sm.pdf]

Science Advances
AAAS

Supplementary Materials for

Predicting evolutionary outcomes through the probability of accessing sequence variants

P. Alexander Gunnarsson and M. Madan Babu

Corresponding author: P. Alexander Gunnarsson, alexgunnarsson90@gmail.com;
M. Madan Babu, madan.babu@stjude.org

Sci. Adv. **9**, eade2903 (2023)
DOI: 10.1126/sciadv.ade2903

This PDF file includes:

Figs. S1 to S7
Tables S1 to S4
Data S1

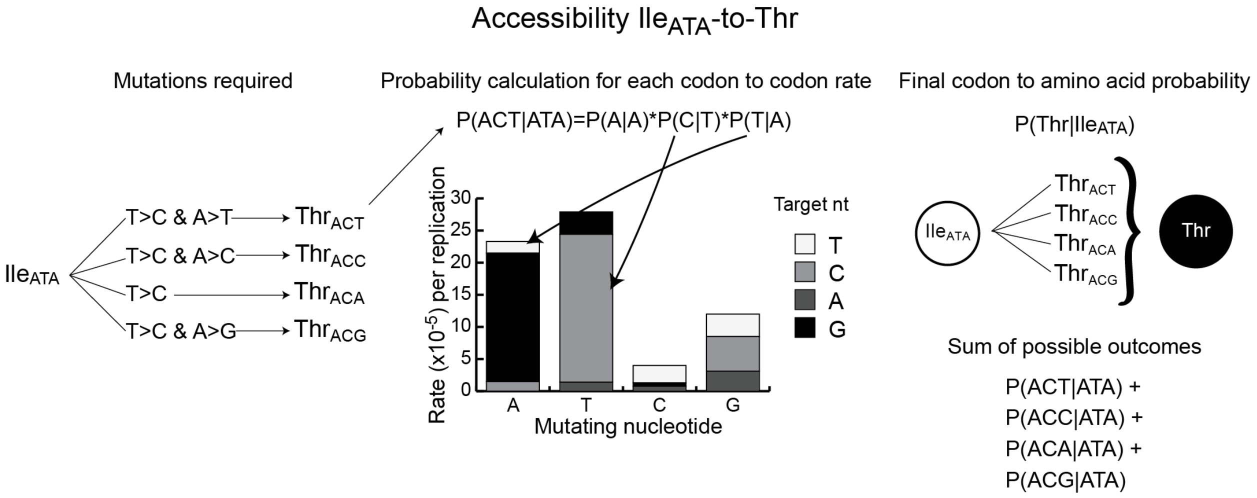

Fig. S1. Calculating amino acid accessibility from Ile$_{ATA}$ to Thr in influenza A.

The probability (P) of reaching any of the threonine (Thr) codons depends on the number of mutations and the identity of the mutation. Because of the biased mutation rate, ATA>ACA has a high probability of occurring.

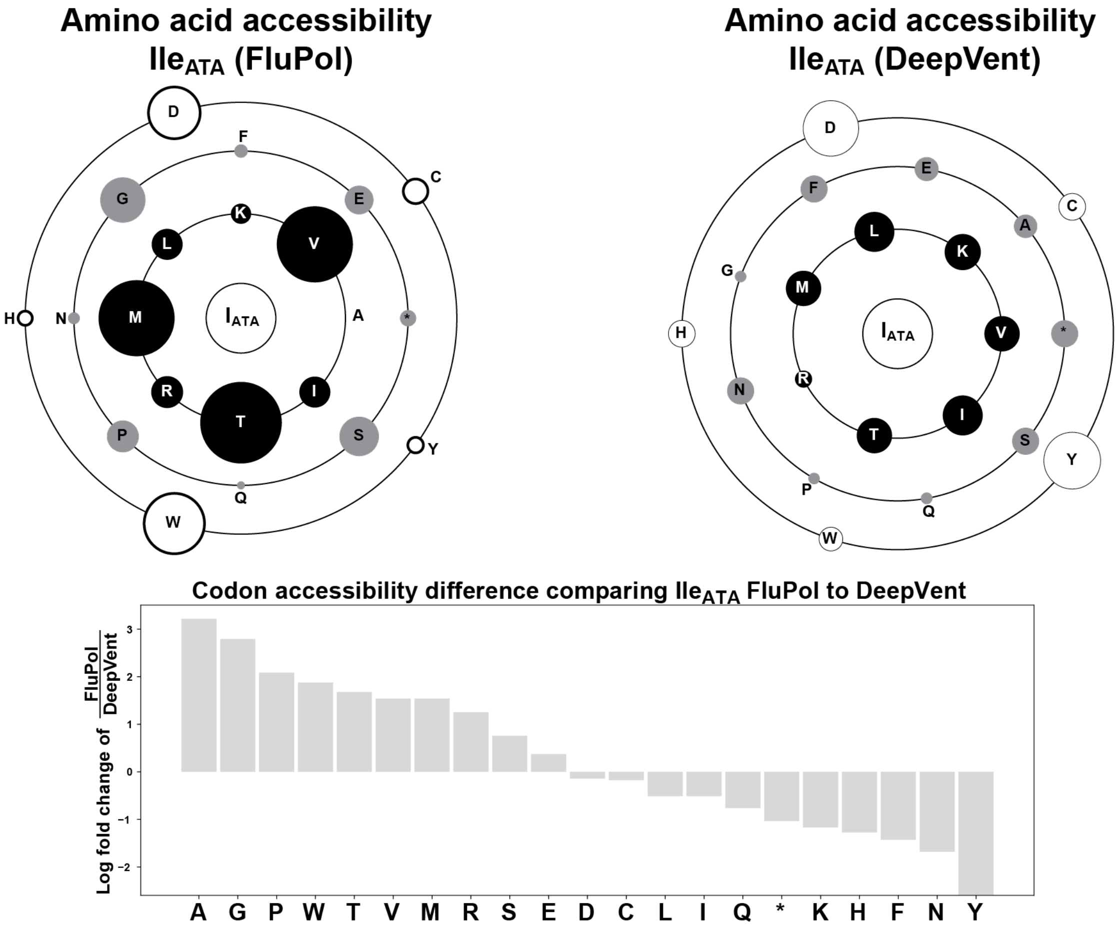

Fig. S2. Accessibility profiles resulting from different polymerases.

On the left is the accessibility profile of Ile$_{ATA}$ mutated with the biases in influenza polymerase. On the right is the accessibility profile of Ile$_{ATA}$ if mutated by DeepVent (exo-), a commercially available polymerase. This highlights the big difference in accessibility depending on the mutational biases in the evolving system. The plot below shows the log ratio of the accessibility of all the amino acids showing that most amino acids are several times more accessible from Ile$_{ATA}$ in one of the backgrounds, e.g., alanine (A) in the influenza polymerase background, and tyrosine (Y) in the DeepVent background.

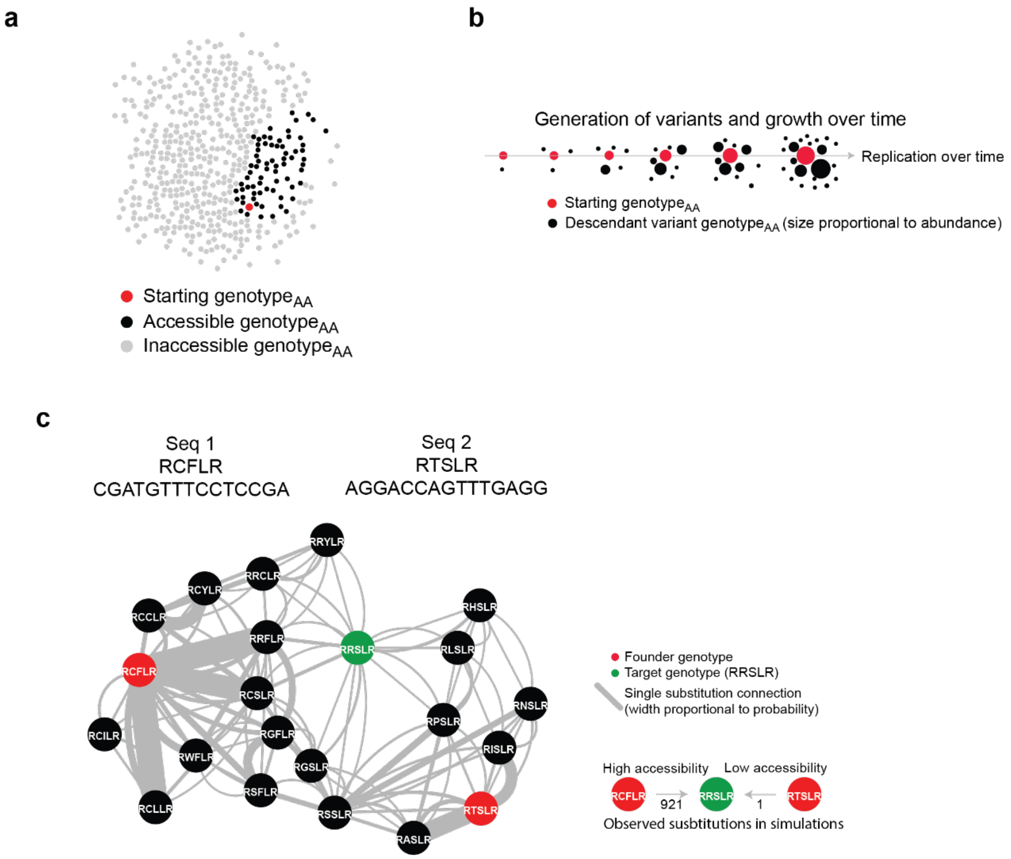

Fig. S3. Accessibility and exploration of variant space.

(**A**) Visualising the accessible part of a potential variant space that defines the variants that can emerge through mutation in the time frame considered. However, this does not highlight the relative likelihood of the different variants emerging. (**B**) As a sequence replicates, new variants emerge through mutation. The more accessible variants emerge early and start growing. Variants that emerge early make up a large subpopulation that is more likely to result in adaptive evolution. (**C**) Visualising how accessibility biases the paths taken through variant space and the consequent likelihood of reaching a particular target variant. Here two starting points, RCFLR and RTSLR, are compared. The target considered is RRSLR. Seq 1 needs two amino acid substitutions (through two nucleotide mutations). Seq 2 needs one amino acid substitution (through two nucleotide mutations). Due to the mutational biases (indicated by thicker connecting lines), Seq 1 has a much higher likelihood of sampling the target variant, because the pathway is much more accessible. This could not be surmised if mutation rate biases and codon choices were not factored in. On the surface, a single amino acid substitution would be expected to be more likely than two amino acid substitutions, but in this example, the opposite would be true.

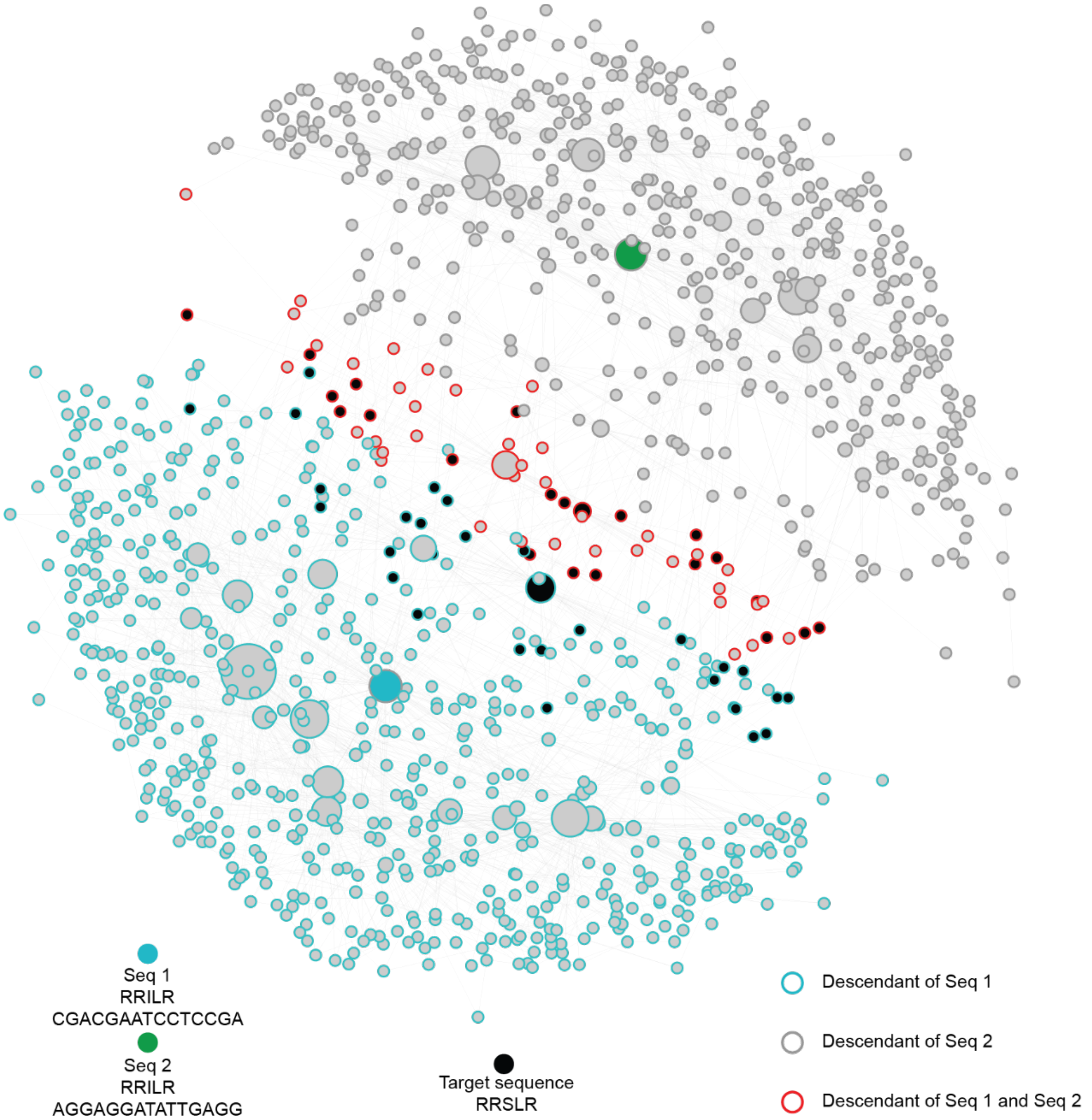

Fig. S4. Variant nucleotide space and evolvability during simulations of two synonymous sequences using IAV mutation rate biases.

Visualizing the shared genotype space of two populations, starting from the two sequences simulated in **Fig 3B**. Despite identical amino acid sequences, the different synonymous codon choices result in different variants being sampled. This visualization shows the few shared amino acid sequences in red outline. The target sequence (black) is much more readily accessed by Seq 1, as it has many more paths to the different variants encoding RRSLR. Sequences derived from Seq 1 through mutation have a blue outline, and sequences derived from Seq 2 have a dark grey outline.

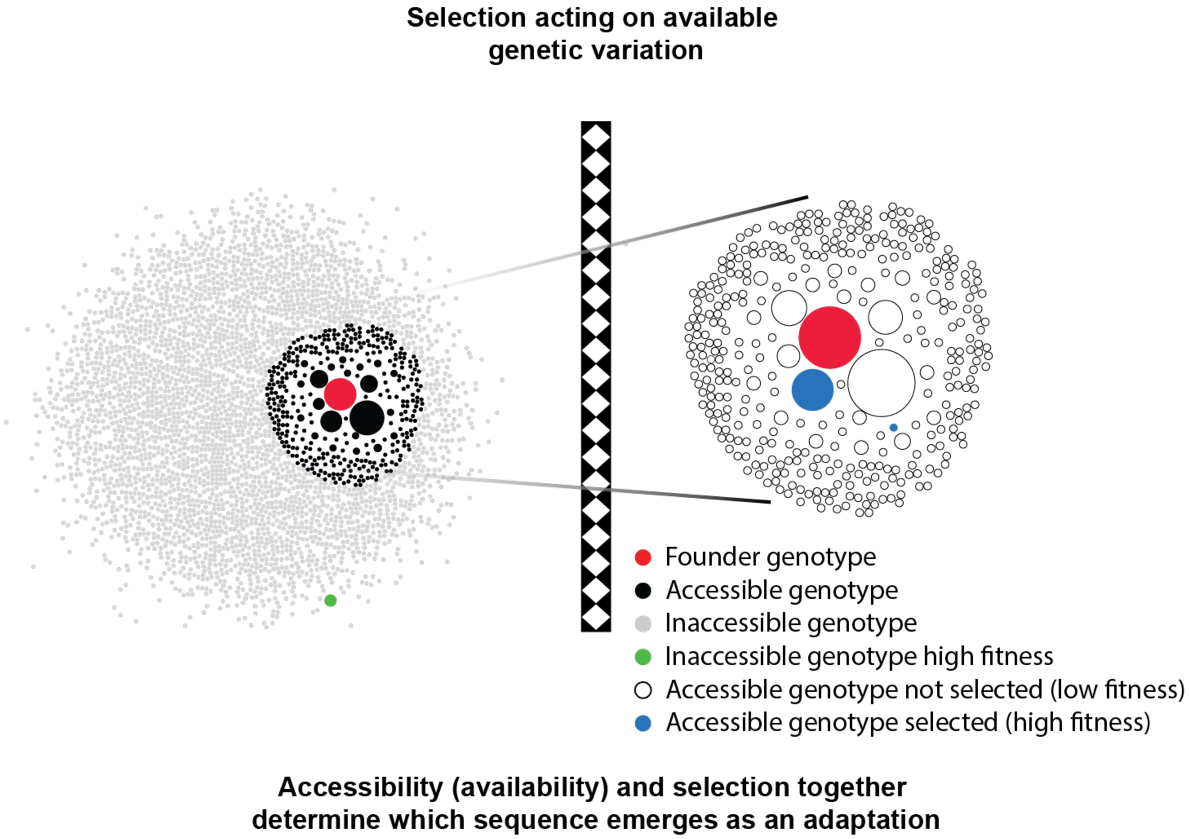

Fig. S5. Selection acts on available variation.

The variation that is generated is what selection can operate on and that can get fixed in a population. This example illustrates a starting genotype sampling sequence space and generating populations of new variants that can be selected. Selection acts on the variants that are generated through the mutational processes, and favorable variants are selected. If they are accessible, they are more likely to emerge in higher numbers and make up larger sub-populations compared to the WT and alternative genotypes, thus making fixation of new favorable variants more likely.

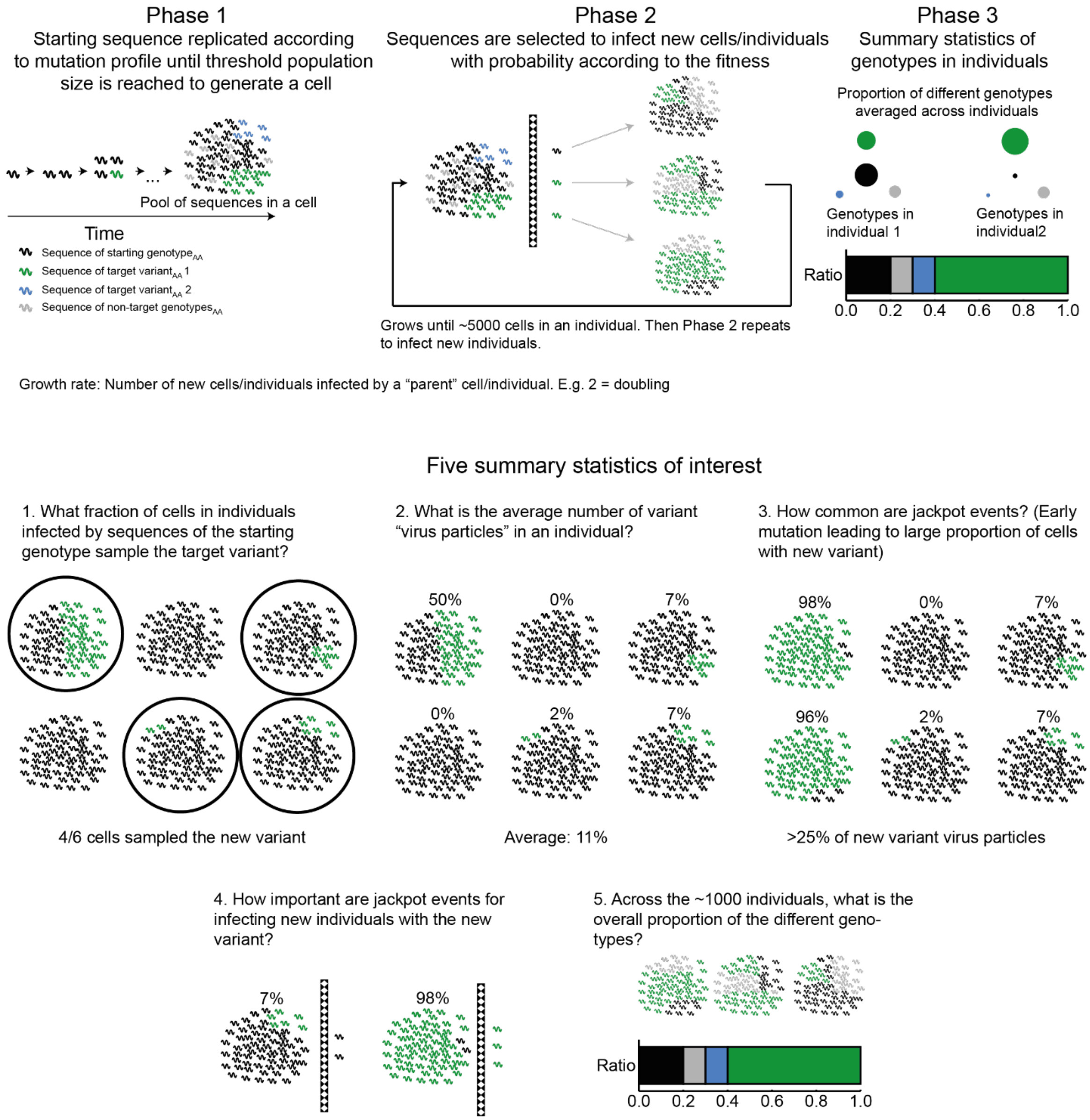

Fig. S6. Simulating viral evolution and infection and the effects of accessibility and fitness on adaptation.

To evaluate the roles of accessibility and fitness together, we simulated sequences with different accessibility and fitness values. We simulated conditions similar to the exponential growth seen in viral infections in three phases, as outlined in the **Methods** and in the figure (top panel). After the simulations, we summarised the results using five metrics that were chosen to reflect aspects of both accessibility and fitness. We quantified how often and how many of each target variant emerged. We also assessed the role of jackpot events and the fixation of new variants in the population.

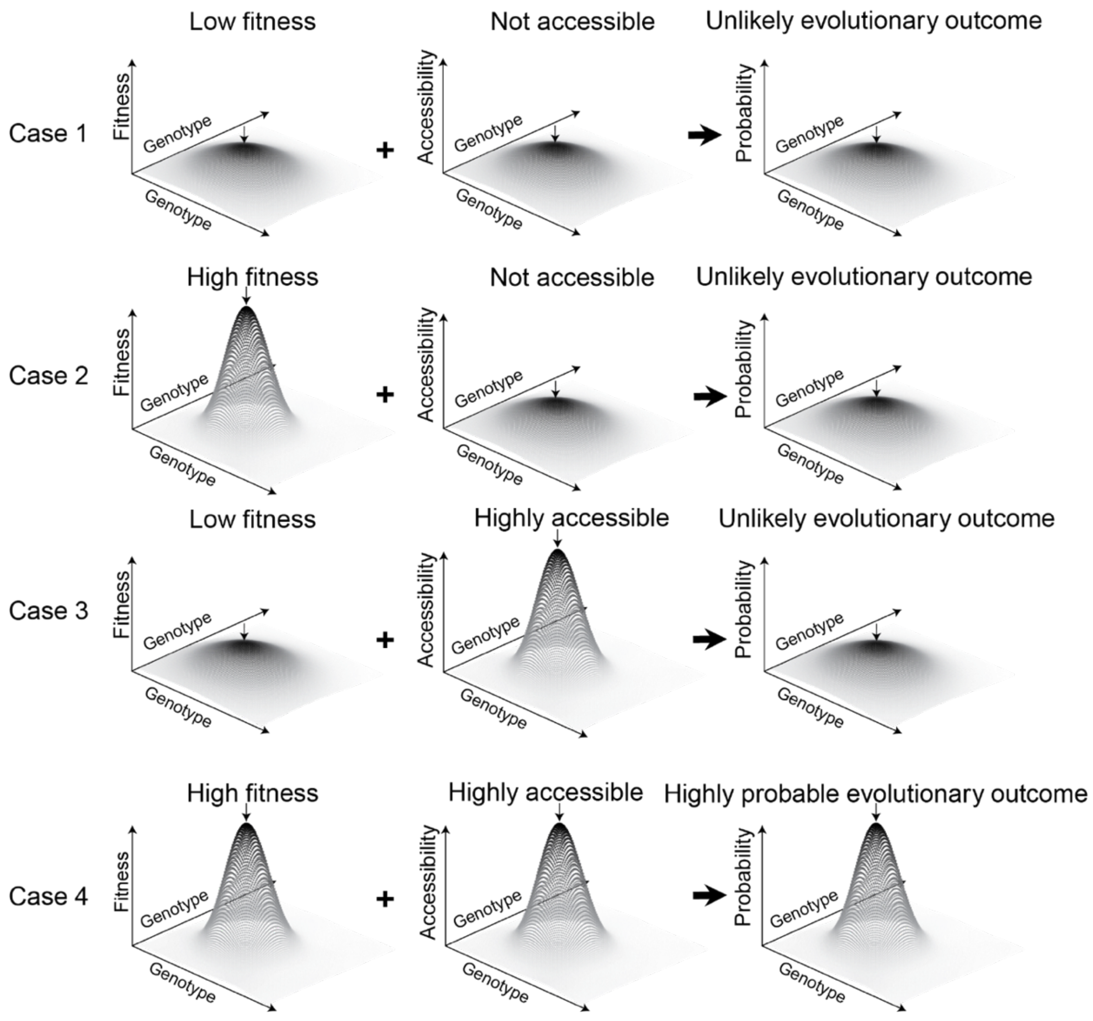

Fig. S7. The effect of accessibility and fitness in the prediction of evolutionary outcomes. Conceptually we can consider the outcomes of 4 different variants. In Case 1, the variant has low fitness and is not accessible. Such a variant is very unlikely to evolve in a population. In Case 2, fitness is high, but since the variant is not accessible, it will be highly unlikely to emerge for selection to act on. Similarly, in Case 3 the variant emerges, however as it is not fit, it will be selected against and will again be unlikely to evolve as an adaptation in a population. Only when sequences are both fit, and highly accessible, as in Case 4, will they be highly likely evolutionary outcomes. In nature, accessibility and fitness will mostly be balanced between these extremes. When accessibility and fitness are moderate there is an equilibrium point where accessibility and fitness influence descendant populations equally (**Fig. 4F).**

Table S1. Mutation rates per round of replication determined experimentally in H1N1 (Pauly et al., (*14*))

**Nucleotide substitution**	**Mutation rate H1N1**	**Rank**	**Mutation rate H3N2**	**Rank**
A>C	1.5×10^{-5}	8	3.4×10^{-5}	7
A>G	2.0×10^{-4}	2	3.0×10^{-4}	2
A>T/U	1.8×10^{-5}	7	1.3×10^{-5}	10
C>A	7.7×10^{-6}	10	1.7×10^{-5}	9
C>G	5.1×10^{-6}	11	9.7×10^{-6}	11
C>T/U	2.7×10^{-5}	6	4.6×10^{-5}	5
G>A	3.1×10^{-5}	5	7.2×10^{-5}	3
G>C	5.4×10^{-5}	3	2.8×10^{-5}	8
G>T/U	3.5×10^{-5}	4	6.0×10^{-5}	4
T/U>A	1.4×10^{-5}	9	4.5×10^{-6}	12
T/U>C	2.3×10^{-4}	1	3.1×10^{-4}	1
T/U>G	3.5×10^{-5}	3	3.6×10^{-5}	6

Table S1. Mutation rates per round of replication determined experimentally in H1N1 (Pauly et al., (*14*)).

Table S2. Contingency table showing robust codons are significantly enriched.

Phosphosite	0	1
Type:		
Fragile	231	7
Neutral	269	84
Robust	392	151

Table S3. Contingency table for full dataset.

Phosphosite	0	1
Codon		
ACA	124	28
ACC	71	48
ACG	21	29
ACT	153	25
AGC	88	7
AGT	143	0
TCA	109	16
TCC	66	37
TCG	15	11
TCT	102	41

Table S4. Loss probability of S-T.

Amino Acid	Codon	Loss Probability
T	ACU	0.000279
T	ACC	0.000279
S	UCU	0.000283
S	UCC	0.000283
T	ACA	0.000285
T	ACG	0.000285
S	UCG	0.000291
S	UCA	0.000296
S	AGC	0.000346
S	AGU	0.000353

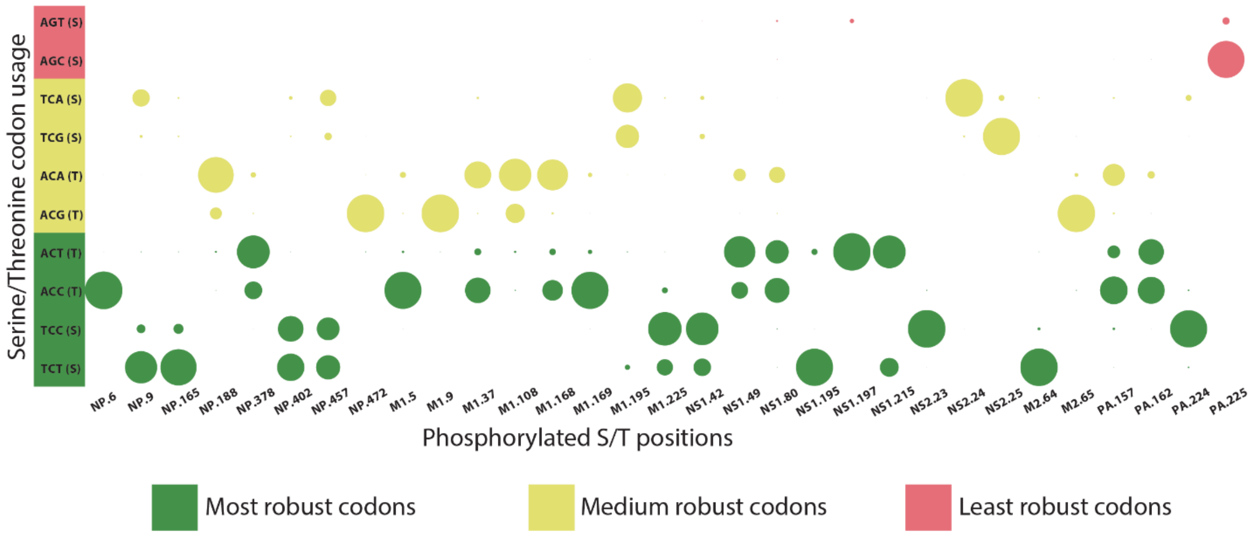

Supplementary Data. 31 literature curated phosphorylation sites used across six proteins in IAV.

This dataset was used for the analysis presented in **Fig. 6**. Size of the circle in the plot represents the frequency of that codon at that site across the different strains analysed (see **Methods**).
